# A mixed methods evaluation assessing the feasibility of implementing a PrEP data dashboard in the Southeastern United States

**DOI:** 10.1186/s12913-023-10451-5

**Published:** 2024-01-18

**Authors:** Kaylee Burgan, C. Greer McCollum, Alfredo Guzman, Brooke Penney, Samantha V. Hill, Kachina Kudroff, Shey Thorn, Toya Burton, Kelly Turner, Michael J. Mugavero, Aadia Rana, Latesha Elopre

**Affiliations:** 1 Division of Infectious Diseases, Heersink School of Medicine, University of Alabama at Birmingham, Birmingham, USA; 2 Department of Pediatrics, Division of Adolescent Medicine, University of Alabama at Birmingham, Birmingham, USA; 3 Five Horizons Health Services, Montgomery, AL 36111 USA; 4 Whatley Health Services, Tuscaloosa, AL 35401 USA; 5 Health Services Center, Hobson City, AL 36201 USA

**Keywords:** PrEP, Pre-exposure prophylaxis, Data dashboard, Care continuum

## Abstract

**Background:**

Alabama is one of seven priority states for the National Ending the HIV Epidemic Initiative due to a disproportionate burden of rural infections. To reverse growing infection rates, the state must increase its focus on prevention efforts, including novel strategies. One such approach is to utilize dashboards that visualize real-time data on the pre-exposure prophylaxis (PrEP) care continuum to assist in prioritizing evidence-based preventative care for those most vulnerable for HIV infection.

**Methods:**

We conducted a mixed methods evaluation to ascertain stakeholders’ perceptions on the acceptability, feasibility, appropriateness, and usability of a PrEP care continuum dashboard, as well as gain insight on ways to improve the activities necessary to sustain it. Clinicians, administrators, and data personnel from participating sites in Alabama completed surveys (*n* = 9) and participated in key informant interviews (*n* = 10) to better understand their experiences with the prototype data dashboard and to share feedback on how it can be modified to best fit their needs.

**Results:**

Surveys and interviews revealed that all participants find the pilot data dashboard to be an acceptable, feasible, and appropriate intervention for clinic use. Overall, stakeholders find the pilot dashboard to be usable and helpful in administrative efforts, such as report and grant writing; however, additional refining is needed in order to reduce burden and optimize usefulness. Participants voiced concerns about their site’s abilities to sustain the dashboard, including the lack of systematized PrEP protocols and limited funds and staff time dedicated to PrEP data collection, cleaning, and upload.

**Conclusion:**

Study participants from clinics providing HIV prevention services, including PrEP, in Alabama voiced interest in sustaining and refining a data dashboard that tracks clients across the PrEP care continuum. Despite viewing the platform itself as an acceptable, feasible, and appropriate intervention, participants agreed that efforts need to be focused on standardizing PrEP data collection protocols in order to ensure consistent, accurate data capture and that limited funds and staff time are barriers to the sustained implementation of the dashboard in practice.

**Supplementary Information:**

The online version contains supplementary material available at 10.1186/s12913-023-10451-5.

## Background

In recent years, nationally coordinated efforts, such as the National HIV/AIDS Strategy and Ending the HIV Epidemic (EHE), have strived to prioritize HIV prevention in regions with high prevalence, specifically through effective biomedical interventions such as HIV pre-exposure prophylaxis (PrEP). Alabama (AL) is one of seven priority states for EHE efforts due to it facing a significant burden of rural HIV diagnoses, as well as various social and economic disparities that disproportionately impact populations vulnerable to infection. In order to more effectively address increasing HIV infection rates, priority must be placed on prevention efforts, such as through identifying and filling gaps in prevention service provision [1, 2].

In the last few years, public data management and visualization systems, such as AIDSVu, have been used to guide and evaluate public health programs, and allow diverse stakeholders the opportunity to engage with, understand, and use data to inform interventions and policymaking [3]. While such platforms have focused heavily on existing population-based HIV data, there is a need to track client-level data across the PrEP care continuum, which would provide real-time, actionable data to community providers and federal agencies attempting to implement evidence-based interventions aimed at reducing HIV incidence.

Data dashboards allow clinicians and managers the ability to visualize and explore data on care processes and outcomes, making them a powerful and supportive tool in decision-making [4]. Dashboards are used in healthcare settings to monitor a variety of healthcare issues, including patient safety, vaccine benefit-risk analysis, quality improvement, opioid overdoses, and inter-departmental coordination [5–9]. Although effectiveness research on data dashboards in HIV care is somewhat limited, recent studies have demonstrated that dashboards hold the potential to increase public access to HIV epidemiological data, enable researchers’ access to HIV data, and enhance clinical care [3, 10, 11]. After implementing health department-based HIV data dashboards in 2011, for instance, New York City saw a 1% increase in linkage-to-care and 16% increase in the percentage of HIV patients with viral load suppression among participating clinics by 2016 [10, 11]. Moreso, the past few years have demonstrated the utility of dashboards in communicating the real-time status of the COVID-19 pandemic, although preliminary studies indicate that the majority of dashboards developed for COVID-19 are only somewhat actionable, primarily due to a lack of focus on identifying specific target audiences [12, 13].

Despite the promise that data dashboards hold in improving HIV care, little is known about how to effectively implement dashboards in clinical settings. Findings from a recent European Union-funded project demonstrate that numerous challenges arise when trying to implement performance dashboards in healthcare settings, including divergent stakeholder expectations; the tension between providing meaningful, accurate, and timely data; and differing dashboard needs and purposes [14]. Other studies have shown that the success of a data dashboard intervention also hinges on the willingness of a site to invest in the intervention financially and with human resources, as well as on the ability of the dashboard to meet certain site requirements. Such requirements include customization capabilities, which would enable users to change display options to best suit preferences, and reporting capabilities, which would allow users to generate visual reports in various formats for site, state, and federal reporting requirements [7, 15]. Studies of other healthcare tools, like electronic health records (EHRs), air-cleaning technology, and smartphone apps reveal similar implementation challenges [16–19]. Notably, though, despite these barriers, many novel technologies, such as EHRs, have widely been adopted in healthcare setting, suggesting the same may be possible for dashboard use in HIV care [20, 21].

Our project, PrOTECT AL (**Pr**EP **O**ptimization **T**hrough **E**nhanced **C**ontinuum **T**racking), is a multiphase, participatory study to coalesce community and public health partnerships in an effort to visualize the state’s PrEP care continuum and provide data visualization via a public-facing data dashboard, a new technology in this space. Building on successful preliminary work, we piloted a dashboard that provided PrEP clinicians, state health officials, and community-based organizations aggregate-level data visualizations on persons engaged in PrEP care across the state. The objective of this study is to better understand the perspectives of key stakeholders, including providers, data personnel, and clinic managers, on the feasibility, acceptability, appropriateness, and usability of the prototype PrEP data dashboard, as well as assess what additional modifications are needed to enhance platform usability, ensure adoption, and facilitate implementation. We believe this project will lay the necessary groundwork for future identification of gaps in the PrEP care continuum.

## Methods

**Fig. 1:**
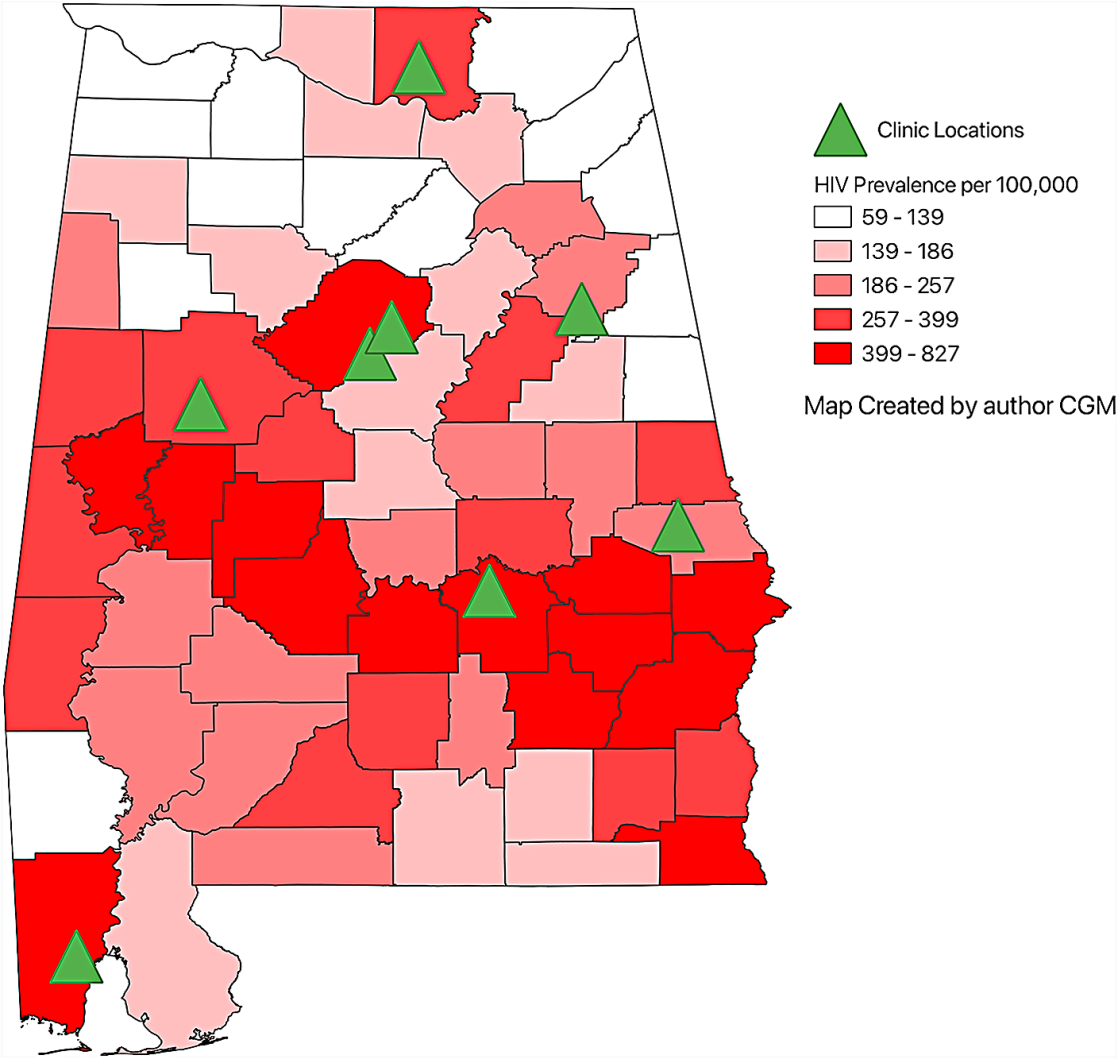
Map of HIV Prevalence in Alabama and participating Ryan White HIV/AIDS Program Clinics [3]. Created with QGIS [24]

The current study was a prospective investigation using a mixed methods approach to evaluate key stakeholders’ perceptions on the feasibility, acceptability, appropriateness, and usability of the pilot PrEP data dashboard. Future stages of this study will evaluate the ability of the dashboard to provide policymakers and providers with actionable data to identify and mitigate any gaps in services. Participation in PrOTECT AL was offered to members of the Alabama Quality Management Group (AQMG), a consortium of 13 clinics who receive Ryan White HIV/AIDS parts C and D program funding in AL. Ten sites expressed interest in participation, but over the course of the study, two sites were unable to continue engagement due to lack of staff and competing priorities. Thus, eight sites from across the state participated in the project: AIDS AL South (Mobile), Health Services Center (Anniston), Five Horizons Health Services (formerly called Medical Advocacy and Outreach in Montgomery), Thrive Alabama (Huntsville), UAB Family Clinic (Birmingham), the UAB 1917 Clinic (Birmingham), Unity Wellness Center (Opelika), and Whatley Health Services (Tuscaloosa). These sites provide a variety of HIV treatment and prevention services and are geographically dispersed throughout the state (Fig. 1). While these sites receive Ryan White funding for patients living with HIV, per the Ryan White Care Act Policy, the use of these funds for PrEP medications and related preventative services is limited [22]. Despite these limitations, the clinics’ PrEP care footprint within the state is quite large in that they provide almost half of all PrEP care across the entire state of AL [3]. Therefore, prior to this study, our investigative team, in collaboration with AQMG partners and the state health department, engaged in an implementation mapping process to better understand how we could leverage PrEP data captured across AQMG organizations to identify service delivery gaps in real-time. The PrOTECT AL data dashboard was felt to be the key implementation strategy necessary to critically visualize and assess real-time data that would promote PrEP prescription [23].

Using findings from surveys, interviews, and focus group discussions in earlier iterations of the study, the research team developed a pilot version of a PrEP data dashboard and a corresponding clinic-facing website [23]. To develop this dashboard, our partners completed a test data upload that corresponded with an agreed-upon data codebook developed via focus group discussions; this upload included three months of clinic data on clients seeking STI/HIV testing services and PrEP clients. Data Use Agreements (DUAs) with each site were established prior to data submission and detailed the agreed-upon data elements to be collected. Clinics were provided with a Data Management Protocol, a document designed to guide participating sites through the goals and responsibilities for participation, as well as detail the upload process. Sites were also given a final copy of the data dictionary with the elements necessary for upload and an upload template that would allow for accurate, consistent data entry for the development of the dashboard. Clinics were given four weeks to compile and upload this data. Six of the eight PrOTECT AL partners submitted clean, usable data for this project.

### PrOTECT AL dashboard features

**Fig. 2:**
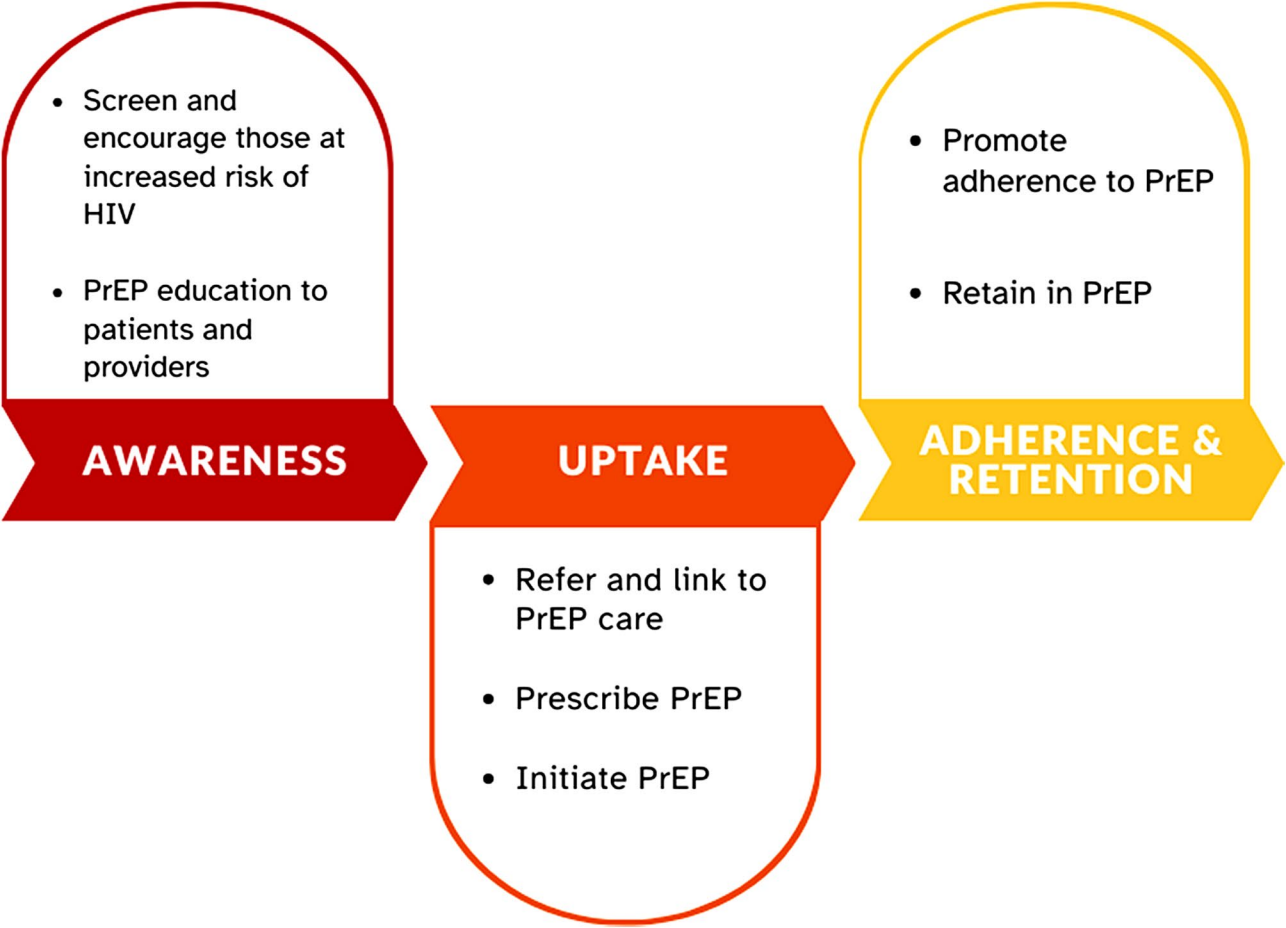
Continuum of PrEP Care. Modified from CDC [25]

The PrOTECT AL website consists of a public-facing homepage with information on the purpose of the project, resources for finding PrEP across the state, information on project partners, and reading materials on HIV and prevention efforts across AL. From the website, partners can access the data dashboard via a clinic-specific login where they can view both aggregate data and their clinic-level data. As the goal of the dashboard is to visualize the PrEP Care Continuum for participating sites in Alabama, as described by the CDC’s Continuum of PrEP Care (Fig. 2), data elements include, but are not limited to: number of patients referred for, linked to, prescribed, or discontinued from PrEP within the reporting period; primary risk factors recorded; and AL counties served. These elements can be filtered down individually by race, age, and gender. A copy of the full data dictionary can be found in Appendix A.

Following development, partners were given approximately two weeks to engage with the dashboard and website to provide feedback on the acceptability and usability of the data dashboard, as well as perceptions on the feasibility of adopting this dashboard in their clinic.

### AIM, IAM, FIM survey and SUS survey

Following development and engagement with the pilot PrEP data dashboard, 10 stakeholders across the eight participating sites were asked to complete an anonymous survey via Qualtrics, an online survey platform. An overview of participation can be found in Table 1.

The goal of this survey was to measure stakeholders’ perceptions on the acceptability, appropriateness, and feasibility of the PrEP data dashboard, as well as the usability of the platform.

**Table 1:** Overview of participation per site

Site	A	B	C	D	E	F	G	H
Survey (*n*)	1	1	-	1	2	1	1	2
Interview (*n*)	1	1	-	2	2	1	1	2
Clinic Data Submission	✓	✓	-	✓	✓	✓	-	✓

The survey included the Acceptability of Intervention Measure (AIM), Intervention Appropriateness Measure (IAM), and Feasibility of Intervention Measure (FIM), each of which is a four-item measure of implementation outcomes that are often considered “leading indicators” of implementation success [26]. Definitions for these constructs can be found in Table 2 [27]. The survey also included the System Usability Scale (SUS), a ten-item questionnaire to help provide a global view of subjective assessments of the usability of a system [28]. Each of these measures consists of a five-point Likert scale ranging from “Completely/Strongly Disagree (1)” to “Completely/Strongly Agree (5)”.

**Table 2:** Acceptability, appropriateness, and feasibility framework

Construct	Definition
Acceptability	The perception among implementation stakeholders that a given treatment, service, practice, or innovation is agreeable, palatable, or satisfactory
Appropriateness	The perceived fit, relevance, or compatibility of the innovation or evidence-based practice for a given fit of the innovation to address a particular issue or problem
Feasibility	The extent to which a new treatment or an innovation can be successfully used or carried out within a given agency or setting

### Semi-structured interviews

To better understand the acceptability of the intervention, an in-depth interview guide informed by constructs of acceptability articulated by Sekhon, et al., was developed [29]. The Acceptability Framework defines acceptability as a “multi-faceted construct that reflects the extent to which people delivering a healthcare intervention consider it to be appropriate, based on anticipated or experiential cognitive and emotional responses to the intervention” [29]. This framework was chosen because it measures prospective, concurrent, and/or retrospective assessment of acceptability based on an individual stakeholder’s subjective evaluation of the intervention. It is important to note that this framework differs from the AIM, IAM, FIM framework, as proposed by Proctor et al., as it considers “appropriateness” to be part of “acceptability.”

While the interviews primarily focused on the dashboard itself, we also asked about perceptions on the activities and processes necessary to develop and maintain the dashboard. Interview questions also addressed general feelings towards the public-facing PrOTECT AL website.

Interviews were conducted with a purposive sample of key stakeholders from seven of the eight partner sites; a representative from one of the partner sites was unable to be reached to participate in an interview. Stakeholders include administrative staff, data personnel, and providers who have engaged with the pilot version of the data dashboard. Ten semi-structured interviews were conducted from August 8, 2022 to August 30, 2022 lasting an average of 35 min. The interviews were conducted over Zoom by a member of the Evaluation Unit within the Research and Informatics Service Center (RISC). These interviews were recorded on Zoom; transcribed via Rev, an online transcription service; and coded via NVivo Qualitative Data Analysis software.

This study’s protocols, documents, and forms, including a waiver of documentation for informed consent, were approved by the University of Alabama at Birmingham Institutional Review Board (IRB-300,004,157). Informed consent was obtained verbally and recorded as part of the transcript.

### Analysis

Survey responses were scored according to their instrument’s scoring criteria. AIM, IAM, and FIM survey scale values range from one (1) to five (5), with no reverse scoring. Scores are then summed per response and averaged. SUS survey responses are summed and weighted to determine an overall score per response. Final scores can range from 0 to 100, with 0 indicating no usability and 100 indicating perfect usability [24].

Each interview was coded via NVivo Qualitative Data Analysis software by two coders from the Evaluation Unit within RISC. To help inform qualitative analysis, the research team employed deductive analysis and was guided by constructs from the Acceptability Framework: (1) Affective Attitude; (2) Burden; (3) Ethicality; (4) Intervention Coherence; (5) Opportunity Costs; (6) Perceived Effectiveness; and (7) Self-efficacy. Two of the seven codes, Ethicality and Intervention Coherence, were not used in analysis, as they were not applicable to the purpose of this study.

Following initial coding, the research team developed additional subcodes based on recurring findings from the interviews. For example, the subcode *Proposed Modifications & Additions* was added to better grasp stakeholders’ ideas for refining the platform. Given the large volume of references, we added further subcodes to better group ideas around changes or additions to the intervention and intervention activities: aesthetics, data, data collection procedures, features, public-facing information, upload process, and other/miscellaneous. The most common subcodes among these involve proposed modifications or additions to available features on the dashboard and public-facing information. Similarly, to better understand information on Burden and Perceived Effectiveness, we added the subcodes time, personnel, data capture, and intervention materials and good fit, missing or incomplete data, reporting, and visualization, respectively.

**Fig. 3:**
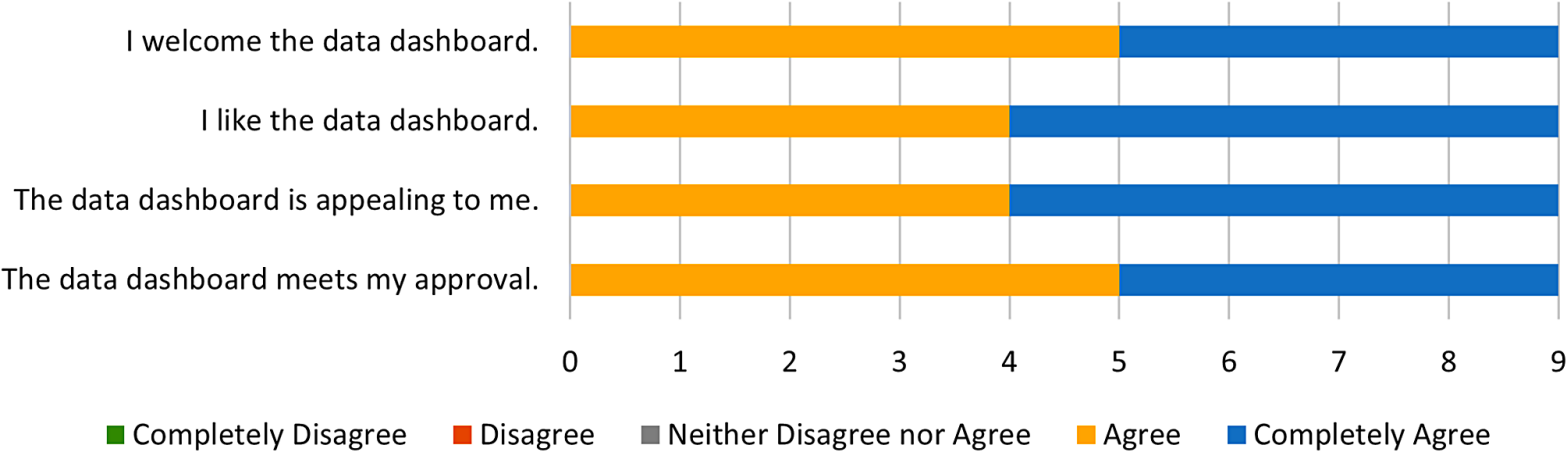
AIM Responses

**Fig. 4:**
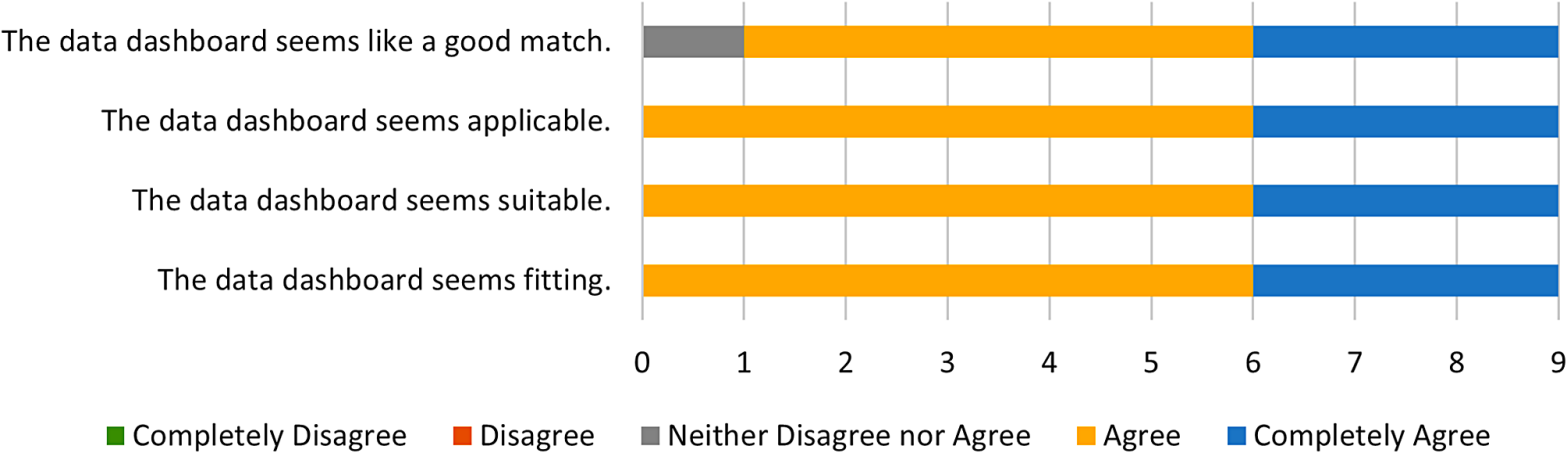
IAM Responses

**Fig. 5:**
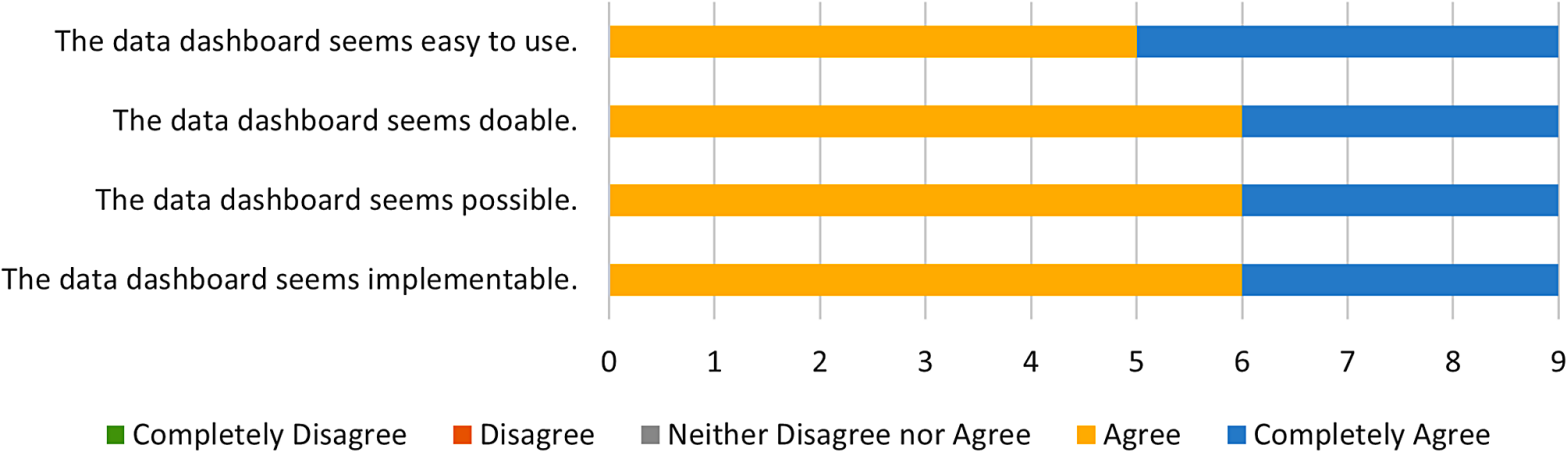
FIM Responses

The coders met three times throughout the analysis process to ensure inter-coder reliability, which is evidenced by a 0.75 kappa coefficient.

Table 3 reflects the analysis framework and corresponding interview questions. A copy of the full interview guide is available in Appendix B. A full list of the codes and subcodes, along with their definitions, can be found in Appendix C.

**Table 3:** Acceptability Framework Constructs and Corresponding Interview Questions

Construct	Definition	Interview Questions
Affective Attitude	How an individual feels about the intervention	• What were your initial thoughts of the dashboard? • Does it contain all the data elements that are most beneficial to your clinic or organization? • Are there any additional data elements you would like displayed? • In what ways can we adjust the data entry and data upload methods to ensure consistent and accurate uploads? • In what ways can we improve the data dashboard to ensure usability in your clinic/organization?
Burden	The perceived amount of effort that is required to participate in the intervention	• Tell me about your experience with data entry for the test data upload for PrOTECT AL. • Tell me about your experience with data upload on the Secure ShareFile link for the test data upload. • Does the data collection for the PrOTECT AL data dashboard fit within your existing workflow?
Opportunity Costs	The extent to which benefits, profits, or values must be given up to engage in the intervention	• What kind of changes to your workflow were necessary to accommodate the dashboard?
Perceived Effectiveness	The extent to which the intervention is perceived as likely to achieve its purpose	• How will the data dashboard be useful to your clinic operations? • How does the data dashboard fit within your clinic’s goals and mission?
Self-Efficacy	The participant’s confidence that they can perform the behavior(s) required to participate in the intervention	• Does the Excel template make sense? Or is there another method that would work best for your workflow? • Does the designated method of upload make sense for your workflow? • How likely will you and your staff adopt or continue to collect data for your site to participate in the PrOTECT AL data dashboard?

## Results

### Survey findings

On the AIM (Mean = 4.5), IAM (M = 4.3), and FIM (M = 4.9) surveys, all participants either Agreed or Completely Agreed with each statement. Only one statement, “The PrOTECT AL Data Dashboard seems to be a good match,” received a mixed response, with one of the nine respondents reporting “neither agree nor disagree.” This indicates that the overwhelming majority of stakeholders find the platform to be acceptable, appropriate, and feasible as an intervention activity. Figures 3 and 4, and 5 show responses to the AIM, IAM, and FIM measures, respectively.

**Fig. 6:**
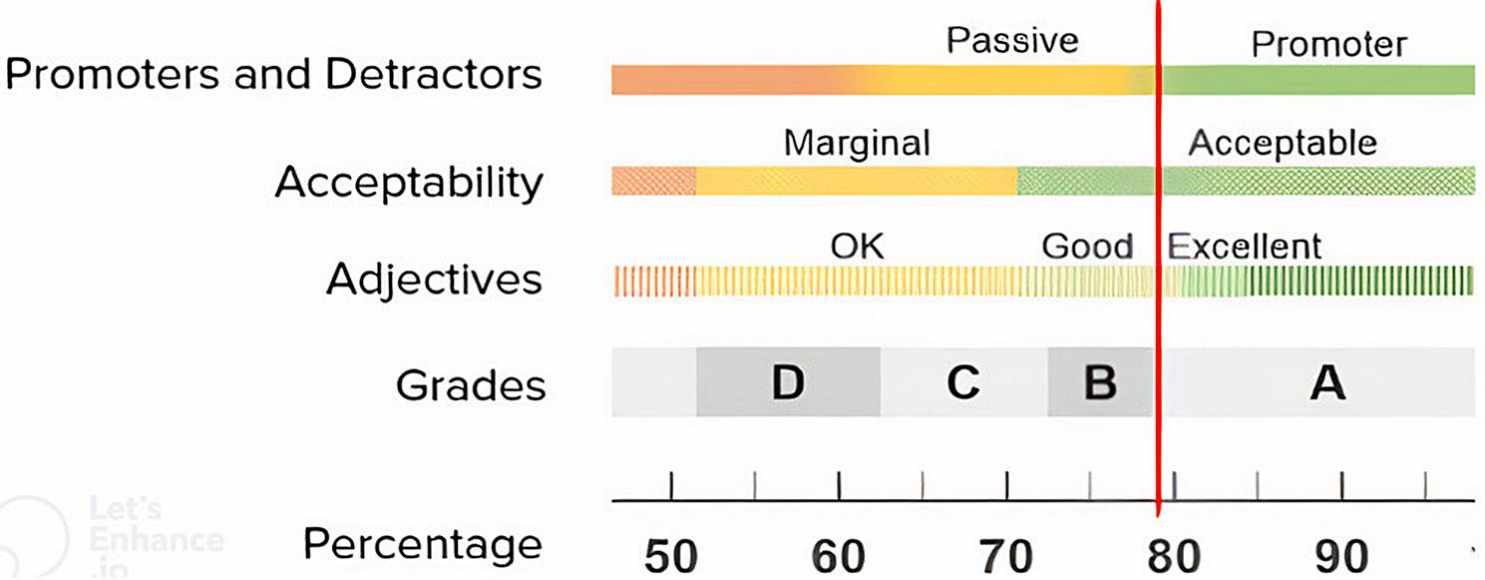
SUS Scoring and Interpretation

According to findings from the SUS survey, the piloted dashboard averaged 79%. According to SUS scoring interpretations, this score, as represented by the red line in Fig. 6, indicates general acceptability and usability by participants. Figure 6 also illustrates the ways in which this score can be interpreted, including the percentile, grade, and adjectives typically used to describe systems of that score. Further, this figure demonstrates that this score is considered “acceptable” by participants and that the dashboard rates as a promoter, meaning that it is likely to be recommended to others by these users.

### Interview findings

During interviews and analysis, we identified several primary and secondary findings. Table 4 summarizes our main findings by Acceptability Framework construct.

### Affective attitude

Overall, participants found the dashboard and corresponding website to be visually appealing, easy to navigate, and a useful tool for clinic operations. One participant noted:

I really like the initiative and the project. I think it’s, you know, good information, not only for the patient, but for us, you know, as clinical staff, helping [and] providing better care to our patients.

However, many participants recommended including additional features to make the dashboard even more useful for clinic operations. Participants seemed most interested in adding options to cross-filter demographic characteristics, such as race, gender, and age group, noting that it would help not only with reporting and applying for grants, but also with sharing information with colleagues in other departments. Nevertheless, participants particularly enjoyed having a quick, simple view of aggregate data and their clinic’s data.

**Table 4:** Coded findings in the Acceptability Framework. Primary findings bolded

Acceptability Framework Construct	Coded Findings
Affective Attitude	• **The pilot dashboard and corresponding website are user friendly, visually appealing, and easy to navigate** (*n*** = 10).** • The dashboard should be refined to provide more cross-filtering options to better assist with reporting, grant applications, and presentations (*n* = 9). • The color scheme is warm and welcoming (*n* = 9).
Perceived Effectiveness	• **The dashboard is or will be useful to clinic operations, specifically in terms of grant and report writing** (*n*** = 10).** • **The data collection and upload process fits well within current clinic workflow** (*n*** = 8).** • Some of the required data elements, such as patient-reported outcomes on food and transportation security, are not consistently collected for PrEP clients (*n* = 7).
Burden	• **All participants** (*n*** = 10) found the intervention activities necessary to develop and maintain the data dashboard to be burdensome.** • Electronic health records (EHRs) makes it difficult to collect some data elements (*n* = 6). • Some participants (*n* = 3) had to use personal time to locate, collate, and upload the required data elements for the pilot. • **Similarly, several** (*n*** = 6) reported that they did not have staff available to do this extensive data reporting.**
Self-Efficacy	• **Participants** (*n*** = 9) reported feeling confident in their ability to complete the tasks necessary to participate in the data dashboard; however, further refinement of this workflow is needed.** • Regularly scheduled deadlines for uploads help with planning and preparation, which would increase confidence in the validity of reported data (*n* = 8).

Overall, participants found the public-facing website to be very clean, clear, and visually appealing as a user and viewer; however, many felt it was lacking more useful information, not only for clinicians but for potential PrEP users, as well. One participant stated, “The resources page, I just felt like it needed more information,” and recommended including frameworks and information outside of EHE objectives. Similarly, two other participants expressed interest in adding the full CDC recommendations for PrEP on either the front-facing page or behind the dashboard login.

The majority of participants found the color scheme of the website and dashboard to be warm and welcoming, however, one participant noted that the abundance of red “subconsciously signals bad or incorrect or wrong.”

When asked about additional modifications or features they would like to see on the dashboard, participants suggested: adding a heat map to the dashboard to better visualize PrEP hotspots across the state; creating a page to house project-related documentation, such as the upload template, codebook, and data management protocol; providing asynchronous trainings that can be accessed on-demand; creating a PrEP working group with regularly occurring meetings; creating a standard template for data collection across sites; and developing a less cumbersome upload template.

### Perceived effectiveness

When asked if they thought the dashboard would be useful to their clinic’s operations, one participant stated:

It absolutely 100% has already proven to be beneficial. So, I know that it would be [even better] […] if the dashboard was expanded to include the information that y’all gathered from all these other sites and we were finally able to implement this on like a policy level or, you know, a clinic level, so that it was something that was within our clinic flow that we knew that we were doing and capturing and continuing to work on. I literally do not foresee any negatives. I only see positives for the grant writing, for better understanding our community and our clients, for having more in-depth conversations with those clients—making them feel more comfortable to be able to talk to us and to share that information—and, honestly, just better understanding where PrEP stands and where we are at as far as the progression of PrEP within our community.

Similarly, another participant noted:

I think it’d certainly be helpful with reporting to, you know, be able to compare and contrast like this is what we know locally versus what the data we’ve gathered kind of more statewide. But also, I think, for education and advocacy, it’s really great to know those numbers.

Further, participants found the dashboard useful in advocating and educating the community on PrEP, with one participant stating:

How can we make PrEP important? So, you know, I think those numbers, they go beyond clinical care, they go, you know, to advocacy, to education and, I think, really could be used to make a difference. ‘Cuz, you know, I have a friend who says you don’t count if you’re not counted. So, if we don’t count PrEP, if we don’t look at this data, it doesn’t matter, and we need it to matter.

Despite the majority of participants finding the dashboard itself to be a good fit and useful to their clinics, many noted that the intervention activities, such as the data collection and upload process, did not fit into clinic operations and workflow very easily. Many participants, particularly data personnel who held primary responsibility for gathering, organizing, and uploading this data, found the process cumbersome and time-consuming as each clinic has a different protocol for handling and collecting data on PrEP patients.

Participants also noted that clinic staff do not consistently capture all data elements, such as patient-reported outcome (PRO) data, which include questions on food security, transportation security, and housing status. Therefore, despite agreeing to and expressing interest in the dashboard displaying PRO data, such data is difficult, if not impossible, to accurately provide for upload to the dashboard. Despite these issues, once sites captured what data they could, they found the upload process via ShareFile, a secure content collaboration and file-sharing software, to be simple and straightforward.

Moreover, while clinics encountered difficulty collecting this data, many noted that it speaks to the greater issue of a lack of federal focus on prioritizing prevention efforts to address the HIV epidemic. One participant noted:

This has been one of my biggest complaints I will say. And concerns just like with our general HIV workforce in the state of Alabama, is that our education hub…I feel like sometimes fails us. And then our state health department, same thing. And I think anybody would generally feel that way if they worked directly with them and saw kind of some of the inconsistencies…you know, this is the dashboard you can use to… we need, we find, we have comprehensive sexual health education approved in the state of Alabama. We need a curriculum for that that we’re all using, not just the schools. So many things just like that, that I think should, for those of us that have been doing this for so long, we should truly have an end all be all to this by now. And we just don’t and I think this is a true testament to that.

### Burden

When asked about burden incurred throughout the study, participants seemed most concerned about which data elements they were and were not able to capture for PrOTECT, as well as how they had to go about collecting all the necessary data. One interviewee described the difficulties, saying:

I think for probably most people, the most difficult piece is, like, gathering the data and then, like, getting it into the right format, and I don’t know that there’s anything that you all could do because of the fact that where everybody’s getting their information from is different.

Similarly, many participants found collating the data into the provided upload template was cumbersome, with one participant noting, “I think because your document was so long, Excel was probably not ideal. Yeah, your codebook was so long.”

Many interviewees described how their clinic’s EHR makes it difficult to collect certain data elements; they have to search different areas of their EHR to collect the information (e.g., provider notes), and, depending on the capabilities of the system, they may have to request assistance from their vendor. Interviewees typically didn’t blame the study’s protocol for these difficulties but accepted them as inevitable, with one participant stating, “I don’t know a better way either. You know, like sometimes there are just things you have to deal with and overcome because there is no better way.”

An adjacent burden interviewees ran into with the intervention was the time it took to collect and upload data to the dashboard. One person explained:

This took up a lot of my own personal time as I was that main point of contact. And I was jumping back through serology forms or the EHR, whatever it may be, to gather this patient information and to put it all together.

Even though interviewees found the intervention to be somewhat burdensome in regard to data capture and time requirements, most agreed they would continue with the intervention because of the benefits it provided, like data for grant writing. One interviewee explained:

It was helpful for me, but it was just overall, it was just time consuming is what it was. And some things, you know, that deserve the time, we’ll get the time. And the things that really will turn out to be beneficial to us and that you put the time into, then it’s all worth it.

Another provider described in a bit more detail why the intervention is worth the burden to them:

So, I like the HIV testing information we gather, anyways, because I use it for different grants and stuff that we turn those numbers in [for]. So for me, I would have no issue continuing to report that information. That would not be difficult for me to do it all.

Despite the barriers faced while trying to implement the intervention, and the sometimes limited effectiveness, it appears most participants feel they are adequately able to perform the necessary intervention tasks. Overall, most interviewees felt they were effectively able to capture the necessary data and upload it in a timely manner despite challenges. One interviewee explained:

If it’s something that we need, we just have to figure out a way to capture it properly on our end. I mean, it’s, I guess it’s doable, you just have to find a way.

A couple of participants discussed how, with the competing demands of healthcare provision, having regularly scheduled deadlines for data uploads would allow participants to adequately plan ahead and mitigate any data capture and upload challenges. One interviewee described the best plan for ensuring their site’s continued participation:

My answer is if you continue to tell us that we need data every three months or every quarter, that is gonna be your best bet because, one, that’ll be a reminder to me, like [to] get the data. ‘Cause otherwise it’s like waiting on me to have moments of freedom, which don’t really happen anymore. […] But if you send out the [reminder] every three months or whatever y’all’s timeframe is, I don’t think that’s a problem. I think we could definitely continue and would like to continue adding to the dashboard. I think it’s more so streamlining the way in which we can collect the data.

### Integration of qualitative and quantitative findings

The merging of the data allowed us to identify areas of concordance and discordance between qualitative and quantitative results. Interview and survey data agreed in terms of general acceptance and perceptions of appropriateness, feasibility, and usability of the data dashboard; however, it is important to note that surveys did not explicitly ask participants about their perceptions of the activities and protocols necessary to maintain the data dashboard. While the surveys indicate overwhelmingly positive perceptions, expansive interviews that explore implementation activities reveal that participants encountered significant burden when collating data elements for upload. A display of concordant and discordant findings across surveys and interviews is shown in Table 5.

**Table 5:** Concordance and discordance of quantitative and qualitative findings. Quantitative surveys consist of 5-point Likert scale from “complete disagree (1)” to “completely agree (5)”

	Quantitative Data	Qualitative Data	
	*Concordance – Data Dashboard*		
Feasibility	The dashboard is a feasible intervention to implement (M = 4.9).	The data dashboard is a feasible intervention in practice. Participants note that it is easily accessible and usable by relevant staff.	
Appropriateness	The dashboard is appropriate for clinic use (M = 4.3), with only one participant indicating neutrality on the dashboard being a good match.	The dashboard is appropriate for clinic use. Participants noted that the dashboard helps visualize the PrEP care continuum and is a useful teaching tool.	
Acceptability	The dashboard as a highly acceptable intervention (M = 4.5).	The data dashboard is an acceptable intervention for adoption at their clinic. Participants report that the visuals assist with report and grant writing, as well as comparing their clinic’s PrEP care continuum outcomes to other clinics’.	
	*Discordance – Qualitative Findings on Data Collection and Management*		
Feasibility	This survey measure (FIM) did not directly address perceived feasibility of the activities necessary to maintain the data dashboard. Therefore, survey findings do not demonstrate whether activity changes are needed.		The current activities needed maintain the data dashboard (e.g., data collection and upload) are not feasible in clinic. Also, certain data elements cannot be collected in a systematic fashion. Lack of time, staff availability, and funding were cited as primary barriers to ensuring proper data collection and management.
Acceptability	Surveys (IAM and AIM) assign different meaning to “appropriateness” and “acceptability” [22]. Surveys do not indicate that changes are needed to make the activities more appropriate or acceptable in clinic.		The Acceptability framework [25] used in interviews, however, considers “appropriateness” to be an aspect of “acceptability”. Therefore, interviews did not differentiate “appropriateness” from “acceptability”. Interviews indicate that the current data collection process and protocol needs refinement to reduce burden. Participants noted significant workflow accommodations, such as the use of personal time, were needed in order to collect and upload for the data. Participants also indicate that a lack of staff availability, as well as the dashboard’s focus on prevention efforts, present barriers to the current data management protocol. The use of a standardized template with requested data elements for PrEP encounters, as well as adjusting the requested data elements, would make the data collection process more manageable. Clinics need to refine their EHRs to assist in accessing data on current PrEP clients.
**Conclusions**			
• Surveys and interviews indicate that the dashboard is a feasible intervention, but interview findings provided more granular insights on the data collection and upload burdens. • Due to the lack of time, staff availability, and funding limitations, the intervention is not feasible at this time for most partners; however, given refinements to the activities needed to maintain the dashboard, it could be in the future. • Development of activities to support adequate data capture, entry, and management is key to ensure clinic adoption and use. • Regional and/or nationally coordinated focus on HIV prevention is needed to assist with consistent and relevant data collection for PrEP clients.			

## Discussion

Our findings demonstrate that, across Alabama, the participating Ryan White HIV/AIDS Program-funded clinics are interested and willing to contribute to a PrEP data dashboard. All participating sites found the pilot data dashboard to be feasible, acceptable, and appropriate, and scored the overall system as usable. Further, stakeholders reported that such a dashboard would be useful for clinic operations, specifically in regard to internal and external report writing, clinic presentations, and drafting grant applications. Dashboards are specifically designed to enhance this kind of data communication, as visualization assists with processing and retaining complex information [30, 31]. Public health dashboards have proliferated over the last several years in the wake of the COVID-19 pandemic, but have been used to communicate data for a variety of other diseases and health issues [13]. AIDSVu, for example, demonstrates that data visualization efficiently communicates regional HIV burden and associated risk factors, as well as gaps in HIV service provision [3, 32, 33].

Despite finding the dashboard itself feasible, acceptable, and appropriate, stakeholders in our study found the procedures necessary to further develop and maintain the dashboard to be burdensome. Stakeholders highlighted key limitations within their existing clinic capacity and workflow that would impact adoption of a PrEP data dashboard, most notably the lack of a standardized PrEP protocol. To stakeholders, this protocol would include a statewide process for assessing PrEP need and providing PrEP care, as well as collecting data elements for the data dashboard. For the majority of these clinics, little attention is given to prevention efforts due to lack of funding and staff availability. For instance, due to funding restrictions, these clinics can only use Ryan White funds for patients living with HIV, not for PrEP clients. Additionally, many data elements necessary to build out the data dashboard, such as PRO data, are not captured, or are not captured systematically, at many sites due to limited capacity. Some stakeholders also noted that not every client eligible for PrEP comes in specifically for PrEP screening, thereby making it difficult to initiate PrEP-specific questions or protocols.

While the lack of a standardized protocol for PrEP patients generates obstacles, the data most clinics have available can create their own set of challenges. Specifically, clinics reported how time-consuming and difficult it was to locate relevant data elements in their EHRs; if the items were found, additional time was needed in order to collate and enter these elements into the provided data template for upload, time that many clinics do not have due staffing limitations.

These sorts of challenges are not unique to the implementation of our dashboard. A 2015 scoping review found that one primary barrier to the implementation of hospital performance dashboards is poor data quality, and scholars have argued that dashboard implementation is hospitals will likely be unsuccessful without the appropriate financials, human resource investments, and close collaboration with those who will be using the dashboard in the clinic [7, 34, 35]. Similar implementation challenges exist for other healthcare technologies, as well. For instance, studies have documented that barriers to transitioning from paper records to EHRs include the prohibitive costs, technical concerns, and insufficient time [19, 36, 37]. Despite these challenges, though, EHRs are now used in nearly 80% of office-based physicians and 96% of hospitals, showing that initially disruptive technologies, like dashboards, can eventually become widely-used tools in healthcare [20].

Interestingly, despite expressing the desire for a PrEP data dashboard to expedite identification of gaps in the PrEP care continuum, participants did not discuss this during the course of our study, nor did they address how, or if, the dashboard would change clinic practice. Future exploration is needed to identify how to make a PrEP data dashboard more informative and useful to clinic operations resulting in clinic practice transformation, as well as in efforts to advocate for funding of HIV prevention data collection. Without nation-wide policies (i.e., a national PrEP program) that are far reaching into states with rural epidemics, like AL, new and innovative strategies will continue to be needed to precisely identify gaps allowing for the expedited allocation of public health resources. It is beyond the scope of this current pilot to evaluate the costs, in terms of person-hours, software, and infrastructure support needed to sustain the dashboard; however, it is broadly accepted and hypothesized that to ostensibly end the epidemic, an integrated health systems approach that prioritizes the allocation of services as well as resources based on disease burden will be necessary [38].

Despite the implementation challenges for the PrOTECT AL dashboard, stakeholders in our study reported that, given the proper funding and staff, their clinics would benefit from providing PrEP data and accessing a data dashboard. Visualizing where clients fall on the PrEP care continuum via a data dashboard is a significant first step in in identifying and closing gaps in the PrEP care continuum in AL, which will move us closer to achieving the ultimate goal ending the HIV epidemic.

### Limitations

Our study included a number of limitations. Most importantly, we initiated PrOTECT in March of 2020 in the midst of the COVID-19 pandemic, a time of increased stress and anxiety for healthcare providers. Two clinics were unable to continue involvement due to lack of staff and competing priorities, and two clinics were unable to provide clean, usable data for similar reasons, thus leaving a smaller sample for the pilot. Because many of these clinics have few staff members, many of whom serve multiple roles, there were few eligible stakeholders from whom we could collate and report data. Therefore, we collected surveys and conducted key informant interviews via a convenience sample, which could present sampling bias. Clinic leadership identified stakeholders including PrEP providers, administrative staff, and data personnel. This convenience sample could limit the representativeness of our findings. Lastly, our study was limited by only working with Ryan White-funded clinics in the state. Other clinics who may provide PrEP were not included.

## Conclusions

The US continues to face high rates of HIV diagnoses with disproportionate burden on rural, Southern states, like Alabama. In order to reduce the number of new infections, more attention must be given on scaling-up effective prevention interventions, like PrEP. Health technologies, such as data dashboards, have the potential to identify gaps in service provision across the PrEP continuum, as well as opportunities to implement evidence-based interventions tailored to the local context. In general, our stakeholders agree that our prototype PrEP data dashboard is a feasible, appropriate, acceptable, and usable intervention for better understanding where patients fall on the PrEP care continuum across the state of Alabama and where action is needed to keep those most vulnerable engaged in HIV preventative care. Despite this, stakeholders identified that more focus is needed on standardizing PrEP assessment, care, and data collection in order to ensure consistent, accurate data capture.

### Electronic supplementary material

Below is the link to the electronic supplementary material.

**Supplementary Material 1:** The attached supplementary materials include a copy of the Data Dictionary provided to sites for clinical uploads to the dashboard, a copy of the qualitative interview guide, and a copy of the codebook developed during analysis of qualitative interviews

## Data Availability

All data is stored in a secure location. Data may be shared, without patient identifiers, contingent upon approval by the UAB Institutional Review Board. For inquiries, please contact the corresponding author.

## List of abbreviations

AL: Alabama
EHE: Ending the HIV Epidemic
PrEP: Pre-exposure prophylaxis
PRO: Patient-reported outcomes
EHR: Electronic health record
