# Supplementary material for: A mixed methods evaluation assessing the feasibility of implementing a PrEP data dashboard in the Southeastern United States
Source: BMC Health Serv Res. 2024 Jan 18;24:101. doi: 10.1186/s12913-023-10451-5 (PMC10797978; doi:10.1186/s12913-023-10451-5)
Supplement: Supplementary file 1 — Supplementary Material 1: The attached supplementary materials include a copy of the Data Dictionary provided to sites for clinical uploads to the dashboard, a copy of the qualitative interview guide, and a copy of the codebook developed during analysis of qualitative interviews [file 12913_2023_10451_MOESM1_ESM.docx]

# Appendix A: Data Dictionary

| **Variable Name** | **Variable** | **Response Options** | **Comments/Required?** |
| --- | --- | --- | --- |
| 1. **Demographic Information (Client-Level Data)** | | | |
| **UploadDate** | Date of Data Upload | MM/DD/YYYY | *Required* |
| **Site** | Site name | 1=UAB 1917 Clinic – Birmingham, AL  2=UAB Family Clinic – Birmingham, AL  3=Thrive Alabama – Huntsville, AL  4=Health Services Center – Anniston, AL  5=Medical Advocacy & Outreach – Montgomery, AL  6=AIDS Alabama – Birmingham, AL  7= AIDS Alabama South – Mobile, AL  8=Unity Wellness – Opelika, AL  9=Franklin Primary Health Center – Mobile, AL  10=Whatley Health Services – Tuscaloosa, AL  11=DIS (dropdown for which county) | *Required* |
| **ClientID** | Unique client ID | This is the 9-digit UCI or URN (unencrypted). It is composed of the first and third letters of the client’s first name, the first and third letters of the client’s last name, the month and day of dob (mmdd), and the last digit of birth year. | *Required* |
| Age | Age at Reporting | Enter client age at time of Reporting | *Required* |
| BirthYear | Year of birth | Enter YYYY | *Required* |
| Ethnicity | Ethnicity | 0=Non-Hispanic  1=Hispanic  99=Refused to answer  9= Missing (Unknown) | *Required* |
| Race | Race | 1=White  2=Black or African American  3=Asian  4=Native Hawaiian/Pacific Islander  5=American Indian or Alaska Native  6=Other (including biracial)  99=Refused to answer  9= Missing (Unknown) | *Required*  *For other 6=Other, describe in variable “other race”, which can include a mix of those listed under “Race” or any other description* |
| AsianSub | Asian subgroup | 1=Asian Indian  2=Chinese  3=Filipino  4=Japanese  5=Korean  6=Vietnamese  7=Other Asian  99=Refused to answer  9 = Missing (Unknown) | *Required*  *If Race = Asian, Client’s Asian subgroup.*  *(choose all that apply)* |
| NHPISub | NHPI subgroup | 1=Native Hawaiian  2=Guamanian or Chamorro  3=Samoan  4=Other Pacific Islander  99=Refused to answer  9= Missing (Unknown) | *Required*  *If Race = Native Hawaiian/Pacific Islander, Client’s Native Hawaiian/Pacific Islander subgroup.*  *(choose all that apply)* |
| Gender | Client’s current gender identity | 1=Male  2=Female  3=Nonbinary  4=Transgender Male-to-Female  5=Transgender Female-to-Male  6=Refused to Answer  9=Missing (Unknown) | *Required* |
| SexAtBirth | The sex assigned to the client at birth | 1=Male  2=Female | *Required* |
| HIVRiskFactor | Client’s HIV risk factor | 1=Male who has sex with male(s) (MSM based on sex assigned at birth)  2=Injecting drug use (IDU)  3=Partner living with HIV  4=Heterosexual sex  5= Positive STI in last 6 months  6=Pregnant individuals living with a partner w/ HIV  7=Intimate Partner Violence (IPV)  8=Transactional sex  10=Multiple sexual partners in last 6 months  99=Refused to answer  9= Missing (Unknown) | *Required*  *Choose all that apply* |
| MedIns | Latest Client’s medical insurance | 1=Private - Employer  2=Private - Individual  3=Medicare  4=Medicaid, CHIP or other public plan  5=VA, Tricare, or other military health care  6=IHS (Indian Health service)  7=Other plan  8=No Insurance/uninsured  99=Refused to answer  9= Missing (Unknown) | *Required* |
| HousingStatus | Latest Client’s housing status. | 1=Stable/Permanent (rent or owns)  2=Homeless/Houseless  99=Refused to answer  9= Missing (Unknown) | *Required* |
| Income | Latest Client’s Income (Yearly). | Integer | *Required* |
| IncomeType | Income Type (client income or household income) | 1=Client Income  2=Household Income  99=Refused to answer  9= Missing (Unknown) | *Required* |
| HouseHold | Latest Client’s members in house | Count: Integer | *Required* |
| GeoCode | Client’s first 3 digits of Zip code | Count: Integer | *If available – use either GeoCode OR CountyCode.*  *One of these variables must be present.* |
| CountyCode | Client’s county of residence | 001= Autauga County  002= Baldwin County  003= Barbour County  004= Bibb County  005= Blount County  006= Bullock County  007= Butler County  008= Calhoun County  009= Chambers County  010= Cherokee County  011= Chilton County  012= Choctaw County  013= Clarke County  014= Clay County  015= Cleburne County  016= Coffee County  017= Colbert County  018= Conecuh County  019= Coosa County  020= Covington County  021= Crenshaw County  022= Cullman County  023= Dale County  024= Dallas County  025= De Kalb County  026= Elmore County  027= Escambia County  028= Etowah County  029= Fayette County  030= Franklin County  031= Geneva County  032= Greene County  033= Hale County  034= Henry County  035= Houston County  036= Jackson County  037= Jefferson County  038= Lamar County  039= Lauderdale County  040= Lawrence County  041=Lee County  042= Limestone County  043= Lowndes County  044= Macon County  045= Madison County  046= Marengo County  047= Marion County  048= Marshall County  049= Mobile County  050= Monroe County  051= Montgomery County  052= Morgan County  053= Perry County  054= Pickens County  055= Pike County  056= Randolph County  057= Russell County  058= St. Clair County  059= Shelby County  060= Sumter County  061= Talladega County  062= Tallapoosa County  063= Tuscaloosa County  064= Walker County  065= Washington County  066= Wilcox County  067= Winston County | *If available – use either GeoCode OR CountyCode.*  *One of these variables must be present.* |
| 1. **Lab Information** | | | |
| **Site** | Site name | 1=UAB 1917 Clinic – Birmingham, AL  2=UAB Family Clinic – Birmingham, AL  3=Thrive Alabama – Huntsville, AL  4=Health Services Center – Anniston, AL  5=Medical Advocacy & Outreach – Montgomery, AL  6=AIDS Alabama – Birmingham, AL  7= AIDS Alabama South – Mobile, AL  8=Unity Wellness – Opelika, AL  9=Franklin Primary Health Center – Mobile, AL  10=Whatley Health Services – Tuscaloosa, AL  11=DIS (dropdown for which county) | *Required* |
| **ClientID** | Unique client ID | This is the 9-digit UCI or URN (unencrypted). It is composed of the first and third letters of the client’s first name, the first and third letters of the client’s last name, the month and day of dob (mmdd), and the last digit of birth year. | *Required* |
| STIScreen | STIs client screened for | 1=Chlamydia  2=Genital Herpes  3=Gonorrhea  4=Hepatitis B  5=Herpes  6=HPV (Human Papillomavirus)  7=Syphilis  8=Trichomoniasis  9=Other | *If available*  *Choose all that apply*  *If “9=Other,” allow open text for manual entry.* |
| STIScreenDt | Dates of STI screening in reporting period | MM/DD/YYYY | *If available*  *Must be within the reporting period start and end dates.* |
| STIResults | STIs client tested positive for | 1=Chlamydia  2=Genital Herpes  3=Gonorrhea  4=Hepatitis B  5=Herpes  6=HPV (Human Papillomavirus)  7=Syphilis  8=Trichomoniasis  9=Other | *If available*  *Choose all that apply*  *If “9=Other,” allow open text for manual entry.* |
| HIVTest | Indicate if patient was tested for HIV | 1=Yes  2=No | *If available* |
| HIVTestDate | Date of HIV Test | MM/DD/YYYY | *If available*  *Must be within the reporting period start and end dates.* |
| HIVTestType | Indicate which HIV test was used | 1=Rapid Oral  2=Rapid Blood  3=Lab-based  4=Other | *If available*  *If “4=Other,” allow open text for manual entry.* |
| 1. **PrEP Care Continuum** | | | |
| **PrEP Referral** | | | |
| **Site** | Site name | 1=UAB 1917 Clinic – Birmingham, AL  2=UAB Family Clinic – Birmingham, AL  3=Thrive Alabama – Huntsville, AL  4=Health Services Center – Anniston, AL  5=Medical Advocacy & Outreach – Montgomery, AL  6=AIDS Alabama – Birmingham, AL  7= AIDS Alabama South – Mobile, AL  8=Unity Wellness – Opelika, AL  9=Franklin Primary Health Center – Mobile, AL  10=Whatley Health Services – Tuscaloosa, AL  11=DIS (dropdown for which county) | *Required* |
| **ClientID** | Unique client ID | This is the 9-digit UCI or URN (unencrypted). It is composed of the first and third letters of the client’s first name, the first and third letters of the client’s last name, the month and day of dob (mmdd), and the last digit of birth year. | *Required* |
| PrEPEducation | PrEP education communication or materials (pamphlets, flyers, etc.) given to patient | 1=Yes  2=No | *If available* |
| PrEPScreenDt | Screen date for this client during this report period | MM/DD/YYYY | *If available*  *Must be within the reporting period start and end dates.* |
| PrEPReferral | Indicate if referral (i.e referral to PrEP Clinic) was completed | 1=Yes  2=No | *If available* |
| PrEPReferralDeclined | Indicate reason why patient declined PrEP referral | 1=Not at risk  2=Cost of PrEP  3=Side effects of PrEP  4=Stigma  5=Inability to come to appointments  6=Other | *If available*  *If “6=Other,” allow open text for manual entry.* |
| PrEPReferredDt | Referral date for this client during this report period | MM/DD/YYYY | *If available*  *Must be within the reporting period start and end dates.* |
| PrEPReferralType | Type of referral provided | 1=Warm Handoff  2=Contact info given  3=Other | *If available*  *1=Warm Handoff, provide appointment information* |
| PrEPInitialApptDate | Appointment date for this client during this report period | MM/DD/YYYY | *If available*  *Must be within the reporting period start and end dates.* |
| PrEPReferralSite | Location patient was referred to for PrEP services | 1= AIDS Alabama – Birmingham, AL  2= AIDS Alabama South – Mobile, AL  3= Children’s of Alabama – Birmingham, AL  4= Five Horizons – Tuscaloosa, AL  5= Health Services Center – Anniston, AL  6= MAO – Montgomery, AL  7=Thrive Alabama – Huntsville, AL  8=UAB 1917 Clinic – Birmingham, AL  9=UAB Family Clinic – Birmingham, AL  10=Unity Wellness – Opelika, AL  11=Franklin Primary Health Center – Mobile, AL  12=Whatley Health Services – Tuscaloosa, AL  13=Jefferson County Health Department  14=Mobile County Health Department  15=Other | *If “15=Other,” allow open text for manual entry.* |
| **PrEP Prescription** | | | |
| **Site** | Site name | 1=UAB 1917 Clinic – Birmingham, AL  2=UAB Family Clinic – Birmingham, AL  3=Thrive Alabama – Huntsville, AL  4=Health Services Center – Anniston, AL  5=Medical Advocacy & Outreach – Montgomery, AL  6=AIDS Alabama – Birmingham, AL  7=AIDS Alabama South – Mobile, AL  8=Unity Wellness – Opelika, AL  9=Franklin Primary Health Center – Mobile, AL  10=Whatley Health Services – Tuscaloosa, AL  11=DIS (dropdown to select county) | *Required* |
| **ClientID** | Unique client ID | This is the 9-digit UCI or URN (unencrypted). It is composed of the first and third letters of the client’s first name, the first and third letters of the client’s last name, the month and day of dob (mmdd), and the last digit of birth year. | *Required* |
| PrEPRx | Indicate if a prescription is provided to the client | 1=Yes  2=No | *If available* |
| PrEPRxDeclined | Indicate reason why patient declined PrEP prescription | 1=Not at risk  2=Cost of PrEP  3=Side effects of PrEP  4=Stigma  5=Inability to come to appointments  6=Other | *If available*  *If “6=Other,” allow open text for manual entry.* |
| PrEPRxDt | Prescription date for this client during this report period | MM/DD/YYYY | *Must be within the reporting period start and end dates.* |
| PrEPRxType | Indicating if the prescription received is an initial prescription or a refill. | 1=Initial  2=Refill/Renewal |  |
| **PrEP Discontinued** | | | |
| **Site** | Site name | 1=UAB 1917 Clinic – Birmingham, AL  2=UAB Family Clinic – Birmingham, AL  3=Thrive Alabama – Huntsville, AL  4=Health Services Center – Anniston, AL  5=Medical Advocacy & Outreach – Montgomery, AL  6=AIDS Alabama – Birmingham, AL  7= AIDS Alabama South – Mobile, AL  8=Unity Wellness – Opelika, AL  9=Franklin Primary Health Center – Mobile, AL  10=Whatley Health Services – Tuscaloosa, AL  11=DIS (dropdown for which county) | *Required* |
| **ClientID** | Unique client ID | This is the 9-digit UCI or URN (unencrypted). It is composed of the first and third letters of the client’s first name, the first and third letters of the client’s last name, the month and day of dob (mmdd), and the last digit of birth year. | *Required* |
| PrEPDiscontinued | Indicate if PrEP care is discontinued | 1=Yes  2=No | *If available* |
| PrEPDiscontinuedReason | Indicate reason why patient discontinued PrEP | 1=Not at risk  2=Cost of PrEP  3=Side effects of PrEP  4=Stigma  5=Inability to come to appointments  6=Other | *If available*  *If “6=Other,” allow open text for manual entry.* |
| PrEPDiscontinuedDt | Discontinuation date for this client during this report period | MM/DD/YYYY | *Must be within the reporting period start and end dates.* |
| **PrEP Visit Information** | | | |
| **Site** | Site name | 1=UAB 1917 Clinic – Birmingham, AL  2=UAB Family Clinic – Birmingham, AL  3=Thrive Alabama – Huntsville, AL  4=Health Services Center – Anniston, AL  5=Medical Advocacy & Outreach – Montgomery, AL  6=AIDS Alabama – Birmingham, AL  7= AIDS Alabama South – Mobile, AL  8=Unity Wellness – Opelika, AL  9=Franklin Primary Health Center – Mobile, AL  10=Whatley Health Services – Tuscaloosa, AL  11=DIS (dropdown for which county) | *Required* |
| **ClientID** | Unique client ID | This is the 9-digit UCI or URN (unencrypted). It is composed of the first and third letters of the client’s first name, the first and third letters of the client’s last name, the month and day of dob (mmdd), and the last digit of birth year. | *Required* |
| PrEPVisitDt | PrEP provider appointment date | MM/DD/YYYY | *Must be within the reporting period start and end dates.*  *Do not Include sick calls. Exceptions may apply.* |
| PrEPVisitModality | Modality of patient encounter | 1 =In Person  2 =Video  3 =Telephone | *If available* |
| PrEPVisitStatus | Status of PrEP Care Visit | 1=Arrived  2=No Show  3=Cancelled  4=Rescheduled/Bumped | *If available* |
| 1. **Patient Reported Outcomes (PROs)** | | | |
| Site | Site name | 1=UAB 1917 Clinic – Birmingham, AL  2=UAB Family Clinic – Birmingham, AL  3=Thrive Alabama – Huntsville, AL  4=Health Services Center – Anniston, AL  5=Medical Advocacy & Outreach – Montgomery, AL  6=AIDS Alabama – Birmingham, AL  7= AIDS Alabama South – Mobile, AL  8=Unity Wellness – Opelika, AL  9=Franklin Primary Health Center – Mobile, AL  10=Whatley Health Services – Tuscaloosa, AL  11=DIS (dropdown for which county) | *Required* |
| ClientID | Unique client ID | This is the 9-digit UCI or URN (unencrypted). It is composed of the first and third letters of the client’s first name, the first and third letters of the client’s last name, the month and day of dob (mmdd), and the last digit of birth year. | *Required* |
| PHQInterest | Little interest or pleasure in doing things? | 0 =Not at all  1 =Several days  2 =More than half the days  3 =Nearly every day | *If available* |
| PHQHopeless | Feeling down, depressed, or hopeless? | 0 =Not at all  1 =Several days  2 =More than half the days  3 =Nearly every day | *If available* |
| SubstanceUseLife | Values indicating patient reporting substance use in their lifetime | 1=Yes  2=No  99=Refused to answer  9=Missing information | *If available* |
| SubstanceUse3mo | Values indicating patient reporting substance use in the last 3 months | 1=Yes  2=No  99=Refused to answer  9=Missing information | *If available* |
| FoodInsecure | Values indicating patient reporting food insecurity in the last 12 months | 1=Yes  2=No  99=Refused to answer  9=Missing information | *If available* |
| TransportationSecure | Values indicating patient reporting transportation security in the last 12 months | 1=Yes  2=No  99=Refused to answer  9=Missing information | *If available* |
| TransportModality | Values indicating patient reporting primary mode of transportation | 1=Personal automobile  2=Bike  3=Walking  4=Ridesharing  5=Public transportation (bus, subway, train, etc.)  99=Refused to answer  9=Missing information | *If available* |

# Appendix B. Interview Guide

1. First, can you start by telling me about your role at [clinic or organization name]?
   1. How will you be interacting with the PrOTECT AL data dashboard?
2. Have you had the opportunity to review the PrOTECT AL data dashboard?
   1. If yes, move on.
   2. If no, show data dashboard on screen.
3. What were your initial thoughts of the dashboard?
   1. Does it contain all the data elements that are most beneficial to your clinic or organization?
      1. Are there any additional data elements you would like displayed?
4. Are there any additional features you would like to see on the data dashboard?
5. Tell me about your experience with data entry for the test data upload for PrOTECT AL.
   1. Was the process difficult or confusing?
      1. Are there any aspects we can improve upon?
   2. Does the Excel template make sense? Or is there another method that would work best for your workflow?
   3. What barriers do you encounter in trying to capture these data? What processes need to be in place in order for you to capture these data variables?
   4. What would motivate you to capture these data variables?
6. Tell me about your experience with data upload on the Secure ShareFile link for the test data upload.
   1. Was the process difficult or confusing?
      1. Are there any aspects we can improve upon?
   2. Does that method of upload make sense for your workflow?
7. In what ways can we adjust the data entry and data upload methods to ensure consistent and accurate uploads?
   1. Are there any additional resources we could offer to make this process easier?
8. Does the data collection for the PrOTECT AL data dashboard fit within your existing workflow?
   1. What kind of changes to your workflow were necessary to accommodate the dashboard?
9. How likely will you and your staff adopt or continue to collect data for your site to participate in the PrOTECT AL data dashboard?
   1. How will the data dashboard be useful to your clinic operations?
   2. How does the data dashboard fit within your clinic’s goals and mission?
10. How likely will you and your staff want to use the PrOTECT AL data dashboard?
    1. In what ways can we improve the data dashboard to ensure usability in your clinic/organization?
    2. How many potential users are there in your clinic?
11. Are there any aesthetic edits or modifications to the dashboard that you would like to see?
    1. Color scheme, data elements, visibility, etc.
12. Those are all of my formal questions. Is there anything regarding PrOTECT AL that we did not cover that you would like to discuss?

# Appendix C. Codebook

| **Construct** | **Definition** |
| --- | --- |
| Affective Attitude | How an individual feels about the intervention |
| Proposed Modifications and Additions | Changes an individual feels is necessary to make the intervention more usable, acceptable, and/or feasible |
| Aesthetics | Changes/additions to the visual appearance of the dashboard and/or website |
| Data | Changes/additions to the data collected or displayed |
| Upload Process | Changes/additions to how data is collected and/or uploaded for the dashboard |
| Features | Changes/additions to any dashboard features |
| Front-facing information | Changes/additions to any resources on the front-facing website |
| Data Collection Procedures | Changes/additions to the way sites collect data from patients |
| Other or Miscellaneous | Changes/additions that did not fit into specific categories |
| Burden | The perceived amount of effort that is required to participate in the intervention |
| Time | Mentions of burden related to time to collect, upload, or view data |
| Personnel | Mentions of burden related to clinic personnel (i.e., not enough staff, not a person dedicated to project, etc.) |
| Data capture | Mentions of burden related to data that can and cannot be captured at the clinic-level |
| Intervention materials | Mentions of provided intervention materials being insufficient or unclear |
| Ethicality | The extent to which the intervention has good fit with an individual’s value system |
| Intervention Coherence | The extent to which the participant understands the intervention and how it works |
| Opportunity Costs | The extent to which benefits, profits, or values must be given up to engage in the intervention |
| Perceived Effectiveness | The extent to which the intervention is perceived as likely to achieve its purpose |
| Reporting | Mentions of how intervention could assist with clinic reporting and/or grant writing |
| Visualization | Mentions of how intervention is helpful with visualizing data |
| Good fit | Mentions of how intervention aligns with clinic priorities, missions, and objectives |
| Missing or Incomplete Data | Mentions of data that sites were unable to capture or unreliable data |
| Self-efficacy | The participant’s confidence that they can perform the behavior(s) required to participate in the intervention |
